# A Life‐Threatening Pharyngeal Infection: Lemierre’s Syndrome

**DOI:** 10.1155/carm/5140821

**Published:** 2025-12-17

**Authors:** Alejandro Mario de la Paz-Estrello, Alba Rodríguez-Pérez, Víctor Eugenio Vera-Delgado, María Candelaria Darias-Martín, Edgar Eduardo Pastor-Garnica, Alen García-Rodríguez, Candelaria Martín-González

**Affiliations:** ^1^ Department of Internal Medicine, University Hospital of the Canary Islands, San Cristóbal de La Laguna, Spain; ^2^ Department of Intensive Care Medicine, University Hospital of the Canary Islands, San Cristóbal de La Laguna, Spain; ^3^ Department of Psychiatry, Dermatology and Internal Medicine, University of La Laguna, San Cristóbal de La Laguna, Spain, ull.es

**Keywords:** abscess, anticoagulation, *Fusobacterium necrophorum*, Lemierre’s syndrome, thrombosis

## Abstract

A previously healthy 28‐year‐old woman who developed pharyngeal, pulmonary, and ocular manifestations due to Lemierre’s syndrome is reported. High‐grade fever, severe sore throat, neck pain, and diarrhea were the initial symptoms. Worsening of these symptoms following oral azithromycin therapy prompted the patient to present to our hospital’s emergency department. Physical examination, chest X‐ray, blood tests, and contrast‐enhanced CT scans of the neck and chest revealed enlargement of the left palatine tonsil, thrombosis of the left internal jugular vein, multiple enlarged left cervical lymph nodes, and bilateral pulmonary consolidations, confirming the diagnosis. Admission to the intensive care unit (ICU) was required due to multiorgan failure. A combination of antibiotic therapy, anticoagulation, and surgical drainage of a left palatine tonsillar abscess was essential for a favorable outcome. After a left‐sided pleural empyema was identified, a chest drainage tube was inserted for treatment. Conservative management was chosen for the ocular involvement. This case highlights the importance of maintaining a high index of clinical suspicion in primary care and emergency settings, as well as the early initiation of an appropriate antibiotic regimen, which can improve the prognosis and reduce morbidity and mortality. Its relevance is increasing, as recent guidelines for oropharyngeal infections may not adequately address this condition because of its low incidence.

## 1. Introduction

Lemierre’s syndrome (LS) is characterized by a pharyngeal infection complicated by internal jugular vein thrombophlebitis and distant septic emboli [1], although the primary infection may originate from other sites, including regions outside the neck [2]. It is usually caused by Gram‐negative bacteria such as *Fusobacterium necrophorum* (*F. necrophorum*), but Gram‐positive organisms have also been implicated, albeit less frequently [1, 3]. It is more prevalent in young individuals, and its incidence was higher in the preantibiotic era; however, the current incidence of 14.4 cases per million people aged 14–24 years [2] makes this condition rare. The dissemination and invasion from the primary site of infection to adjacent veins (most commonly the internal jugular vein) is responsible for the high morbidity and mortality rates associated with this disease. Its tropism for vessels, tendency to cause bacteremia, inhibition of the innate immune response, and activation of the coagulation cascade [3] result in the dissemination of multiple septic emboli. Pneumonia and pleural empyema are the most common metastatic infections caused by this syndrome [1], but soft tissue abscesses, pyomyositis, splenic and liver abscesses, osteomyelitis, endocarditis, renal and brain abscesses, and even various forms of ocular involvement may also occur [1, 2, 4]. The cornerstone of treatment is usually the early initiation of antibiotic therapy, but surgical drainage of abscesses and anticoagulation may also be required depending on the clinical scenario [2, 3]. A case‐fatality rate of 2%–9%, an approximate incidence of serious long‐term complications of around 10% [3, 4], the requirement for a multidisciplinary team for diagnosis and treatment [1–5], and its typical presentation in previously healthy individuals make this infection of great interest to the medical community. The case report presented below describes our diagnostic and therapeutic approach in a 28‐year‐old woman who presented to our hospital with this syndrome.

## 2. Case Presentation

We present the case of a 28‐year‐old woman without a significant medical history except for Epstein–Barr virus infection in 2022 and the use of oral contraceptives. The patient was a high school teacher and had previously led a normal life. Two weeks before admission, she developed a mild sore throat that progressed to severe pain associated with a fever of 38°C a few days later. In addition, she noticed a painful swelling on the left side of her neck. The patient decided to go to a healthcare facility, where azithromycin 500 mg once daily was prescribed. Subsequently, she developed abdominal pain with multiple episodes of diarrhea, and the antibiotic was changed to cephalexin 500 mg four times daily, although the patient chose not to take it. The symptoms worsened and the fever intensified, reaching 39°C daily. At that time, the case was attributed to a possible Epstein–Barr virus infection, and only symptomatic treatment was recommended. The symptoms continued to worsen, with progressive swelling of the left cervical region, which increased in size and extended toward the left preauricular area, making mouth opening difficult. The patient developed dyspnea at rest and was subsequently referred to our hospital’s emergency department. Upon admission, oxygen saturation was 100% while receiving oxygen via nasal prongs at a flow rate of 3 L/min. Tachypnea was noted at 26 breaths per minute, and all other vital signs were within normal limits.

**Figure 1:**
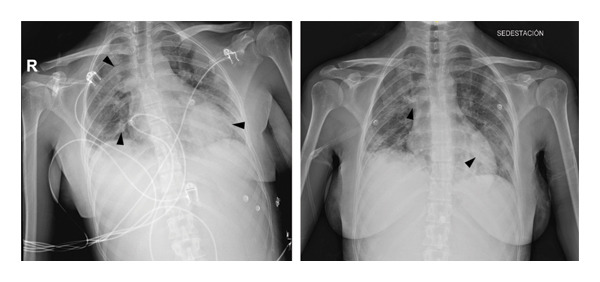
Chest X‐ray in which multiple bilateral pulmonary masses can be seen (black arrows).

**Figure 2:**
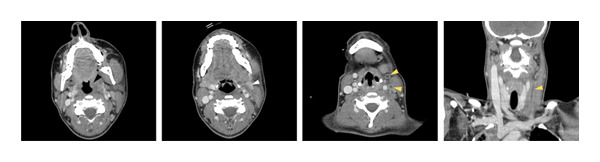
CT‐scan (axial and coronal view) in which enlargement of the left palatine tonsil (black arrow), left internal jugular vein thrombosis (yellow arrows), and left cervical lymph node (white arrow) can be seen.

**Figure 3:**
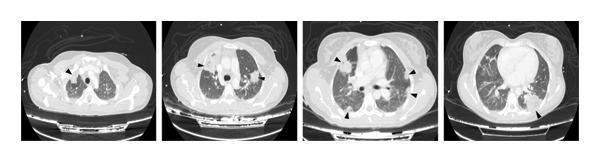
CT‐scan (axial view) in which multiple bilateral lung consolidations can be seen (black arrows).

**Figure 4:**
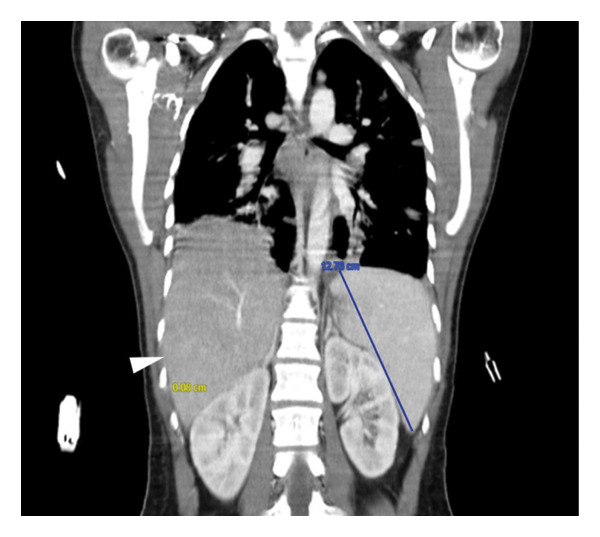
CT‐scan (coronal view) in which hepatosplenomegaly is seen (white arrow and blue mark).

She was bedridden, pale, and in poor general condition. On examination, her left preauricular region was tender and painful to palpation, and multiple enlarged left cervical lymph nodes were palpable. She had difficulty opening her mouth, and the pharynx could not be examined. Lung auscultation revealed bilateral crackles, and no cardiac murmurs were detected. Initial blood tests revealed hemoglobin of 10.1 g/dL, leukocytes 20,920/mm^3^, neutrophils 19,830/mm^3^, platelets 59,000/mm^3^, fibrinogen 1051 mg/dL, creatinine 1.33 mg/dL, procalcitonin 70.9 mg/dL, Interleukin‐6 189 pg/mL, and C‐reactive protein 407.3 mg/dL. Chest X‐rays showed a 5.49 × 5.69‐cm rounded mass in the left lung, a 3.88 × 3.80‐cm right paratracheal rounded mass, and multiple smaller bilateral masses (Figure 1). Blood cultures, multiple viral PCR tests, mumps virus serology, and urinary antigens for *Streptococcus pneumoniae* and *Legionella pneumophila* were collected. At that time, a neck and chest CT scan was ordered with the suspicion of LS and intravenous metronidazole 500 mg three times daily and cefepime 2 g three times daily were started. Enlargement of the left palatine tonsil, left internal jugular vein thrombosis, multiple enlarged left cervical lymph nodes, hepatosplenomegaly, and multiple bilateral lung consolidations were identified, confirming the diagnosis (Figures 2, 3, and 4). The patient was admitted to the ICU for monitoring of multiorgan failure. Seventy‐two hours after admission to the ICU, an exploratory cervicotomy was performed. Under general anesthesia with orotracheal intubation, the patient was positioned supine with right cervical hyperextension. Through a Kocher incision along an upper cervical skin crease, subplatysmal flaps were raised and the cervical fascia and submandibular space exposed, with identification of the anterior belly of the digastric and palpation of the hyoid. The facial and external jugular veins were ligated. Exploration along the anterior border of the sternocleidomastoid muscle revealed indurated tissues and necrotic‐appearing lymphadenopathy; one lymph node was excised for histopathology and microbiology. Dissection proceeded deep to the submandibular gland into the parapharyngeal space, where a small volume of purulent material was drained and sent for culture; the space was decompressed and irrigated copiously with normal saline and rifampicin solution. A left pharyngeal wall dehiscence was repaired. Two drains were left in the parapharyngeal space. Hemostasis was secured, and the wound was closed in layers with skin staples. Drains were removed 72 h postoperatively due to minimal output.

**Figure 5:**
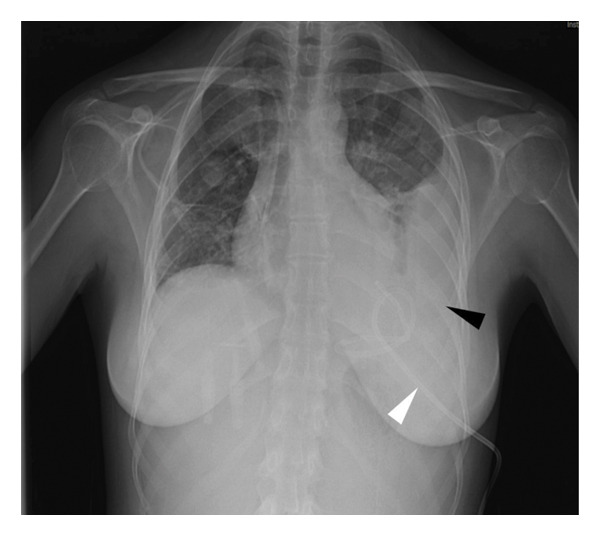
Chest X‐ray in which a left pleural effusion (black arrow) and a left pleural catheter (white arrow) can be seen.

Anticoagulation was started with enoxaparin at a dose of 1.5 mg/kg/day on the sixth day when the platelet count was > 50,000/mm3. It had not been initiated earlier because of severe thrombocytopenia. A cranial CT scan performed several days later demonstrated patency of the venous sinuses. During the ICU stay, the patient experimented diplopia and a left IV cranial nerve’s palsy was diagnosed. In both the blood cultures, *F. necrophorum* was isolated, sensitive to metronidazole and clindamycin (Table 1). It was not isolated in the cultures taken from the cervicotomy. Finally, an antibiotic treatment with metronidazole 500 mg three times daily and ceftriaxone 2 g two times daily was elected. The patient was transferred to the internal medicine unit 9 days after the ICU admission. A transthoracic echocardiography ruled out valve warts. In the 14th day, a left basal lung hypophonesis was noticed and a chest X‐ray confirmed a left pleural effusion (Figure 5). A left‐sided thoracentesis was performed. Pleural fluid analysis showed yellow, turbid appearance; red blood cell count canceled; cell count not performed because of clotting; glucose 21 mg/dL; and total protein 3.6 g/dL. An empyema was confirmed, and a 10‐Fr pleural catheter (Pleurocath) was inserted via a left posterior approach, yielding a small amount of serous fluid, and connected to −20 mmHg suction. Total output was 1250 mL. It was retired after 12 days. Pleural fluid cultures (bacterial and fungal) were negative.

**Table 1:** *Fusobacterium necrophorum’s* antibiogram in blood cultures.

Antibiotic	*F. necrophorum*	MIC
Clindamycin	S	0.032
Metronidazole	S	0.032

*Note: F. necrophorum*, *Fusobacterium necrophorum*; S, susceptible.

Abbreviation: MIC, minimum inhibitory concentration.

**Figure 6:**
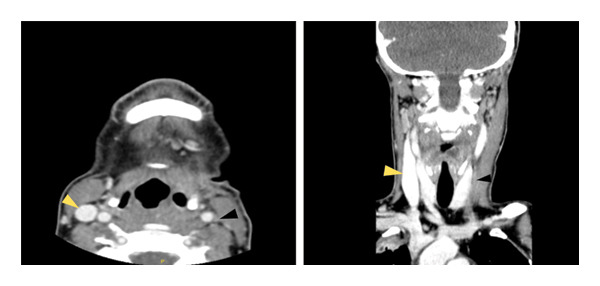
CT‐scan (axial and coronal view) where a left thread‐like internal jugular vein (black arrows) can be seen. The right jugular vein is as well marked (yellow arrows).

**Figure 7:**
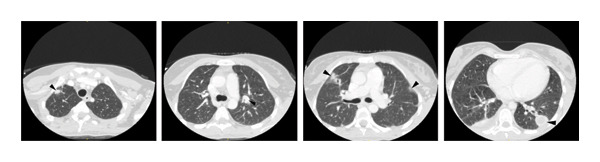
CT‐scan (axial view) in which downsizing of the lung abscesses is observed (black arrows).

The patient improved and was transferred to the home hospitalization unit to complete the last days of antibiotic treatment, although diplopia persisted. A follow‐up CT scan was performed one and a half months after the emergency department visit, which showed a thread‐like left internal jugular vein (Figure 6) and reduction in the size of the lung abscesses (Figure 7). Because of the improvement, ceftriaxone and metronidazole were discontinued, and oral amoxicillin‐clavulanate was prescribed to complete the antibiotic regimen at home. Outpatient follow‐up is currently ongoing.

## 3. Discussion

LS (also known as human necrobacillosis [4]) is defined by postanginal sepsis with internal jugular vein thrombophlebitis and distant septic emboli, most commonly caused by *F. necrophorum*, an oropharyngeal commensal [1, 4]. However, primary infection may arise from other sites, including the pharynx, oral cavity, jaw, ear, and gastrointestinal and genitourinary tracts [2, 6]. Local thrombophlebitis and metastatic septic foci are characteristic features of this syndrome [2].

LS has an annual incidence of 14.4 cases per million among individuals aged 14–24 years and is more common in males (approximately 2:1) [1, 2]. It was relatively common before the antibiotic era and declined after antibiotics were introduced for oropharyngeal infections [2, 4], thereby leading to it being termed “the forgotten disease” [4]. In recent decades, the incidence appears to have increased, potentially related to more restrictive antibiotic use for pharyngitis [2]. Other proposed contributors include increasing antibiotic resistance, changes in prescribing patterns, and reporting trends; suspected Group A streptococcal pharyngitis may also play a role [4]. An association between site of origin and patient age has been described: Oropharyngeal sources predominate in young adults, otomastoiditis in toddlers and children, and head‐and‐neck sources in middle‐aged adults [2].

This syndrome results from bacterial invasion of the pharyngeal mucosa in otherwise healthy individuals or in those with preceding viral or bacterial pharyngitis, leading to septic thrombophlebitis of the internal jugular vein and subsequent metastatic infections [1, 2]. Other primary foci of infection—such as cellulitis, perianal abscess, or septic pelvic thrombophlebitis—have also been described as triggers [2, 7]. The pathogens typically involved are Gram‐negative anaerobes, most commonly *F. necrophorum*, followed by *Fusobacterium nucleatum (F. nucleatum)* and *Bacteroides fragilis (B. fragilis)* [1, 2]; streptococci and staphylococci have also been reported [3]. Failure to isolate *F. necrophorum* may reflect false‐negative culture results, prior antibiotic exposure, or the prolonged incubation required for anaerobes [2]. *F necrophorum* is highly prevalent in the oropharynx of healthy individuals and may be the second most commonly isolated bacterial pathogen in patients with sore throat [2, 3].

The routes by which *F. necrophorum* reaches the internal jugular vein include spread via the tonsillar vein, dissemination through the lymphatic system, or direct extension from peritonsillar abscesses [2]. The organism exhibits vascular tropism, causing bacteremia, suppressing the innate immune response, and directly activating the coagulation cascade [3]. It may breach the oropharyngeal mucosa because of trauma, inflammation, or tissue destruction and subsequently disseminate hematogenously or via lymphatics from a focal abscess to produce sepsis [4]. Once the internal jugular vein or other cervical veins are involved, *F. necrophorum* promotes platelet activation, coagulation, and inflammation, culminating in thrombus formation and subsequent involvement of distant organs [4]. It has virulence factors such as adhesins, endotoxins, hemolysins, and leukotoxins which are implicated in the formation of necrotic abscesses [4]. Immunocompromised patients, oropharyngeal trauma, thrombophilias, hypercoagulable states, specific microorganisms, and environmental conditions constitute recognized risk factors [1, 6, 8]. However, in a Swedish ambispective study conducted in 2015 by Holm et al., no apparent correlation was found between thromboembolic events in LS and underlying thrombophilia. Although *F. necrophorum* has been associated with infectious mononucleosis, this may reflect coinfection or a higher incidence in young adults [4]. No clear association with Epstein–Barr virus has been demonstrated, although viral infection might facilitate bacterial invasion [8].

LS has been compared to endocarditis because of its clinical similarities [3]. A classic triad has been described, comprising pharyngotonsillitis, internal jugular vein thrombosis, and metastatic abscesses, although internal jugular vein thrombosis is not mandatory for the diagnosis [4]. In our patient, the early clinical manifestations coincided with the typical presentation of the disease, including high fever, sore throat, odynophagia, and neck pain with tenderness. In more advanced stages, sepsis, septic shock, and internal jugular vein thrombophlebitis may develop, and by this point, the primary infection may already have resolved [2]. Neck symptoms are often mistaken for lymphadenitis or peritonsillar abscess [2]. Distant complications can represent the initial signs of this syndrome. In our patient, bilateral lung abscesses were present at the time of admission, but the diagnosis of the empyema was established on the 14th day. Pneumonia associated with pleural empyema is known to be the most frequent metastatic infections related to this syndrome [1, 2]. Joints are typically the second most common site of infection dissemination [2, 4]. Other complications may include soft‐tissue abscesses, pyomyositis, splenic and liver abscesses, osteomyelitis, endocarditis, and renal or cerebral abscesses [1, 2].

Central nervous system involvement may present as a meningitis, encephalitis, cavernous sinus thrombosis, or cerebral infarction [2]. Ophthalmologic manifestations are particularly rare and atypical [4, 5]. They result from the transport of septic emboli through the veins of the head and neck [4]. These manifestations include proptosis, orbitopathy, reduced visual acuity, and impaired extraocular motility, usually leading to unilateral involvement [4]. Extraocular muscle dysfunction—such as diplopia, cranial Nerve III/IV/VI palsy, ophthalmoplegia, and strabismus—represents the most common ophthalmologic presentation [4]. Isolated palsies of the abducens and trochlear nerves are most frequent, as described in our patient. Cerebral venous and cavernous sinus thrombosis, as well as septic emboli, may underlie these oculomotor nerve palsies [4, 5]. It has been reported that rates of new or recurrent thromboembolic complications and septic lesions remain high despite hospitalization, and that approximately one in 10 affected patients develops sequelae or requires additional care after discharge [3]. New septic lesions have been described as more frequent than new thromboembolic events [3]. In patients with typical symptoms, the diagnosis is often unclear until general deterioration occurs. LS can be challenging to detect in its early stages because it is clinically indistinguishable from other causes of sore throat and due to its low incidence [2, 4]. A delayed diagnosis of LS may lead to a poor prognosis with multiple complications and even death [1, 4]. Deep neck infection, septicemia, internal jugular vein thrombophlebitis, and ascending or descending septic emboli provide strong evidence for the presence of this syndrome [1]. The general clinical pattern consists of a sore throat with a foul odor, followed by high fever and subsequent jugular vein thrombophlebitis with metastatic lesions [4]. Thrombocytopenia and elevated inflammatory markers may be observed in blood tests [2, 4]. Contrast‐enhanced computed tomography is the diagnostic modality of choice, although Doppler ultrasonography can also assist in the diagnosis, as can magnetic resonance imaging (MRI) in selected cases [1].

Some authors require positive blood cultures for any organism, whereas others specifically require the isolation of *Fusobacterium* species, particularly *F. necrophorum,* for diagnosis [2]. Other authors have argued that there should not be a strict requirement for positive bacterial cultures, since up to 33% of patients may have received antibiotics before sample collection [4]. Radiologically confirmed internal jugular vein thrombosis is also required by some authors for the diagnosis [2]. A recent history of oropharyngeal infection within the previous 4 weeks, along with evidence of metastatic lesions and internal jugular vein thrombosis, has been proposed as the diagnostic criteria [2].

The course of LS is typically rapid and potentially irreversible, and a prompt antibiotic therapy—and occasionally surgical intervention—is mandatory [1, 4]. A multidisciplinary approach is essential [3], usually involving infectious disease specialists, pharmacologists, radiologists, otolaryngologists, ophthalmologists, intensivists, and thoracic surgeons [1, 2, 4, 5]. The treatment usually combines antibiotics with surgical drainage of abscess when necessary. Surgical procedures have been reported in up to 53% of the cases [3], most often in critically ill patients [4]. The most effective antimicrobial regimen consists of metronidazole administered for 3–6 weeks in combination with a β‐lactam antibiotic [1, 2]. It is advisable to use an intravenous route for at least the first 2 weeks [2, 4]. Clindamycin has also been used successfully [3]. In cases of diagnostic uncertainty, empirical broad‐spectrum intravenous antibiotics should be initiated promptly and later refined based on culture results [2]. The clinical response may be slow, likely due to the limited antibiotic penetration into fibrin‐rich thrombi [1].

There is ongoing debate regarding the use of anticoagulation in LS. Some authors believe that infection control alone may lead to thrombus resolution, whereas others express concern that anticoagulation could promote the dissemination of septic emboli [2]. Although early small studies demonstrated no clear benefit, more recent data suggest that anticoagulation may decrease thromboembolic events and appears to be safe [3], showing no increase in bleeding or new septic complications [1]. Anticoagulation has been reported in 23%–56% of cases, typically administered for 70–84 days, but robust evidence supporting its routine use remains limited [2]. In cases of thrombosis involving critical sites such as the cavernous sinus, anticoagulation may play a more significant role [4].

Internal jugular vein thrombosis has been associated with severe infections of the neck—not only with LS but also with central venous catheterization, tumors, intravenous drug use, thrombophilic disorders, and fertility treatments in women [9]. There is limited information regarding the relationship between internal jugular vein thrombosis and oral contraceptive use; however, in two women in Spain in 2006, a possible association was suggested after no additional risk factors were identified following a thrombophilia workup [9]. Also in Spain, internal jugular vein thrombosis associated with upper‐extremity venous thrombosis was described in a 41‐year‐old female smoker taking oral contraceptives [10]. Previous reports have described cases of women with LS and internal jugular vein thrombosis who were taking oral contraceptives, such as the case reported by Tipmongkol et al. in the United States in 2025 [6]. Therefore, in women with LS who are taking oral contraceptives, anticoagulation may be particularly indicated [6].

Regarding ophthalmologic complications of LS, both surgical and nonsurgical management options have been described. For isolated palsies of cranial Nerves III, IV, and VI, occlusive patches, prisms, or botulinum toxin injections may be used in the first 6 months. After this period, the palsy is considered chronic, and corrective surgery becomes the appropriate option [4]. In cases of LS with neurological symptoms suggestive of meningitis, treatment with corticosteroids has been reported, and its discontinuation has been associated with symptom worsening and extension of the thrombosis [5]. In some case reports, corticosteroids appeared to improve the clinical condition [5]; however, there is insufficient scientific evidence to support firm recommendations regarding their use in LS.

The estimated mortality rate ranges from 2% to 9%, although in the preantibiotic era, it was considerably higher [3, 4]. Mortality may increase depending on the timing of antibiotics initiation [2]. The leading cause of death is septic shock, followed by cerebral infarction, intracranial septic embolism, pulmonary embolism, and hemorrhage [3]. The prognosis is poorer in patients who did not receive antibiotic therapy effectively against anaerobes [3]. More than 10% of the survivors experience serious long‐term complications that severely affect quality of life [3] and may develop sequelae such as cranial nerve paralysis, blindness, paralysis, paresis, and other functional impairments [2].

## 4. Conclusion

Clinicians should suspect LS in septic patients with a sore throat, persistent neck pain, or metastatic infections. Early multidisciplinary management, including prompt antibiotic therapy and, in some cases, surgical drainage, is crucial for achieving favorable outcomes. Close follow‐up of patients with a nonimproving sore throat and a negative Group A *Streptococcus* test can help prevent delayed diagnosis.

**Nomenclature**
*B. fragilis*
: *Bacteroides fragilis*
CT: Computed tomography
*F. necrophorum*
: *Fusobacterium necrophorum*
*F. nucleatum*
: *Fusobacterium nucleatum*
ICU: Intensive care unit
LS: Lemierre’s syndrome
MIC: Minimum inhibitory concentration
MRI: Magnetic resonance imaging

## Ethics Statement

The case report meets the ethical guidelines and adheres to the local legal requirements.

## Consent

Written informed consent was obtained from the patient for publication of this case report and any accompanying images.

## Conflicts of Interest

The authors declare no conflicts of interest.

## Funding

No funding was received for this manuscript.
